# Current Treatment Standards for Metastatic Uveal Melanoma

**DOI:** 10.3390/cancers18030475

**Published:** 2026-01-31

**Authors:** Paweł Rogala, Anna M. Czarnecka, Monika Dudzisz-Śledź, Anna Dawidowska, Kacper J. Piwowarek, Piotr Rutkowski

**Affiliations:** 1 Department of Soft Tissue/Bone Sarcoma and Melanoma, Maria Sklodowska-Curie National Research Institute of Oncology, 02-781 Warsaw, Poland; annamalgorzata.czarnecka@nio.gov.pl (A.M.C.); monika.dudzisz-sledz@nio.gov.pl (M.D.-Ś.); piotr.rutkowski@nio.gov.pl (P.R.); 2 Department of Outpatient Chemotherapy, Maria Sklodowska-Curie National Research Institute of Oncology, 02-781 Warsaw, Poland; kacper.piwowarek@nio.gov.pl; 3 Department of Experimental Pharmacology, Mossakowski Medical Research Institute, Polish Academy of Sciences, 02-106 Warsaw, Poland; 4 Early Phase Clinical Trials Unit, Maria Sklodowska-Curie National Research Institute of Oncology, 02-781 Warsaw, Poland; anna.dawidowska@nio.gov.pl

**Keywords:** uveal melanoma, metastases, liver-directed therapy, immunotherapy, tebentafusp, anti-PD1, anti-CTLA4

## Abstract

Background/Objectives: Uveal melanoma (UM) is the most common primary intraocular malignancy in adults, most commonly arising in the choroid. Its development is associated with phenotypic characteristics, ultraviolet radiation, and germline or somatic genetic alterations. Despite progress in diagnostics and local therapies, UM remains characterized by high metastatic risk and poor overall prognosis. This review aimed to summarize current knowledge on epidemiology, clinical features, genetic background, prognostic factors, and therapeutic approaches in metastatic UM. Methods: A structured literature review was conducted to evaluate epidemiological trends, genetic alterations, prognostic markers, clinical presentation, and therapeutic strategies. The results of different systemic treatments were analyzed, with special attention to liver-directed interventions and emerging systemic therapies. Results: The incidence of UM in Europe increases with latitude, ranging from two per million in the southern regions to more than eight per million in the North. The median age at diagnosis is 62 years, and most cases are detected incidentally during ophthalmological examinations due to nonspecific symptoms. Some genetic alterations serve as important prognostic indicators. Local treatment consists of globe-preserving procedures, including radiation therapy, surgery, laser therapy, or enucleation, with failure rates between 6.15% and 20.8%. Up to 70% of patients develop distant metastases, predominantly in the liver. Metastatic UM (mUM) carries a poor prognosis, with overall survival ranging from 3 to 30 months. Liver-directed therapies, particularly surgical resection, provide the most favorable outcomes. Systemic therapies demonstrate limited efficacy; however, tebentafusp has shown an overall survival benefit in HLA-A*02:01 (human leukocyte antigen A*02:01)-positive patients. Conclusions: UM is a rare but aggressive malignancy with limited treatment options once metastatic. Liver-directed strategies remain the mainstay of management, while novel systemic approaches, including tebentafusp, represent promising advances. Further research is required to improve survival and expand therapeutic opportunities.

## Simple Summary

Uveal melanoma is the most common primary cancer of the eye in adults, usually beginning in the choroid. It is a rare but aggressive disease with nonspecific symptoms and many cases are discovered incidentally during eye examinations. Although effective local therapies, such as radiotherapy, surgery, or enucleation, can control the primary tumor, the risk of metastases remains high, affecting up to 70% of patients. The liver is the most frequent location of distant spreading, and once metastases occur, the prognosis is poor, with survival often less than one year. Liver-directed treatments, particularly surgery, may prolong survival, but systemic therapies are generally limited in efficacy. Recently, tebentafusp, a bispecific protein, has shown a survival benefit in selected patients, representing an important step forward. This article summarizes the current knowledge of UM, including its epidemiology, genetic background, clinical features, treatment options, and perspectives for future therapy.

## 1. Introduction

**Figure 1:**
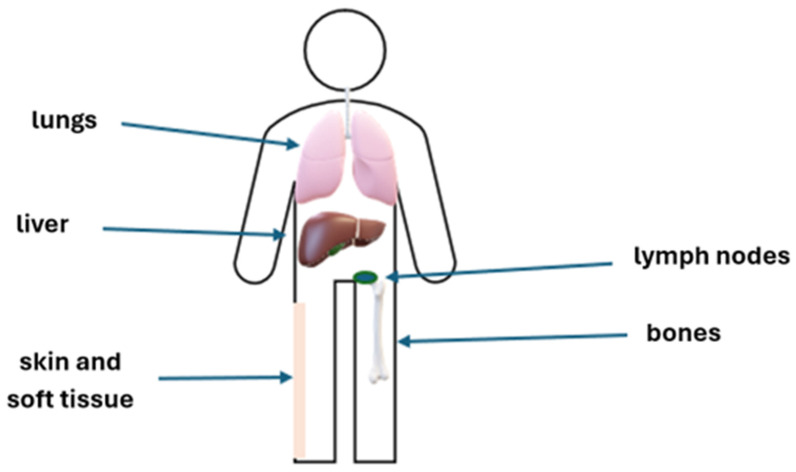
The most frequent sites of UM metastases.

Uveal melanoma (UM) is a rare disease, but it is the most common ocular malignancy in adults [1]. UM arises from melanocytes in the choroid (90%), ciliary body (6%), or iris (4%). This malignancy, which develops from ocular melanocytes, significantly differs from skin melanomas in terms of biology, clinical features, pathogenesis, molecular abnormalities, and clinical course, including the pattern of metastases [2]. The risk factors include light eye color, fair skin, ocular and oculodermal melanocytosis, cutaneous, iris, or choroidal nevi, sunlight exposure to ultraviolet radiation, and mutations in *BRCA1* and BAP1 [3]. Additionally, mutations in the *GNA11*, *GNAQ*, *SF3B1*, and *EIF1AX* genes are often detected in UM. Moreover, mutations in the promoter of *BRAF*, *NRAS*, and *TERT* genes have been described [3,4]. Congenital or infantile UM are rare, and this malignancy mainly develops in adults [5], and most often presents between 50 and 70 years of age, with a median age of 62 at diagnosis. UM is usually unilateral. The incidence worldwide is 1–9/100,000, increases with latitude, and ranges from 2/10^6^ in Spain, to 4–6/10^6^ in Central Europe, to above 8/10^6^ in Denmark and Norway [6]. In about half of the patients, this disease is asymptomatic [7]. The signs and symptoms of UM are nonspecific and include myodesopsia (seeing flying flies), photopsia, high intraocular pressure, or even loss of vision. UMs are most often diagnosed incidentally during ophthalmological examinations. Mutations in *TETR*, *BAP1*, *SF3B1*, and *EIF1AX* are of prognostic significance [8]. Management of localized UM includes either eye-preserving treatment strategies or enucleation. Eye-preserving approaches encompass surgical interventions, radiation therapy, and laser-based modalities. The average treatment failure in all radiation therapies is 6.15%, 18.6% in surgical therapies, and 20.8% in laser therapies [9]. Kaplan–Meier analyses of metastasis-free survival in UM demonstrate age-dependent differences. At 3, 5, 10, and 20 years, the estimated rates are 2%, 9%, 9%, and 20% in younger patients; 6%, 12%, 23%, and 34% among middle-aged adults; and increase to 11%, 19%, 28%, and 39% in elderly individuals [10]. Distant metastases develop in up to 70% of patients. The risk of metastases primarily depends on the stage of the primary tumor and its molecular profile [10,11,12,13,14]. The most frequent sites of metastasis are shown on Figure 1. Metastatic UM (mUM) is a disease with a poor prognosis. The expected overall survival (OS) in the metastatic setting ranges from 3 to 12 months. Based on systematic reviews, meta-analyses, and retrospective studies, the best outcomes are observed in patients treated with a liver-directed approach, in particular, complete resection [15,16,17,18]. Systemic therapies, which are of limited efficacy, may be considered if liver-directed therapies are not feasible. Tebentafusp, a bispecific protein, has been approved for the treatment of patients with mUM with HLA-A*02:01 (human leukocyte antigen A*02:01)-positive disease, based on improved OS [19]. The study aimed to review current and prospective approaches in metastatic ocular melanoma. 

## 2. Risk Stratification and Surveillance of Extraocular Disease in UM

At the time of diagnosis, approximately 97% of patients with UM are limited to the eye, and proper treatment prevents local recurrence in about 87% of these patients. Only 3% of patients are diagnosed in the metastatic stage at the time of presentation. Patients who have undergone treatment for primary tumors should undergo ophthalmological examinations every 3–6 months for the first 2–5 years (duration of follow-up every 3–6 months differs between regions and countries), and every 6–12 months thereafter to detect potential local recurrence or treatment complications [20,21,22,23]. Due to the risk of dissemination, the patients should also be properly monitored to detect and treat any potential metastases. Up to 70% of patients after treatment of localized UM will develop distant metastases within 20 years. The main risk factors are primary tumor size and genetic characteristics. The liver is the most frequent site of metastases, but these may also develop in other organs, such as the lungs, bones, skin, soft tissue, and lymph nodes. The recommended follow-up is based on the assessment of the risk of metastases, which is mostly associated with the stage of ocular disease (stage T assessed based on AJCC, American Joint Committee on Cancer) and molecular abnormalities (Table 1). If liver metastases are suspected, liver MRI (magnetic resonance imaging) with contrast is recommended. Liver function tests can also be performed, but their value is limited due to their low sensitivity [24,25]. Additional imaging tests, including CT (computed tomography) with contrast and PET (positron emission tomography), should be performed if necessary; however, neither CT with contrast nor PET is sensitive enough to replace liver MRI. There is no need to perform a brain MRI unless the patient has signs and/or symptoms of brain metastases, but it may be considered after 2 years of metastatic disease [26]. For extrahepatic metastases, the recommended imaging modalities are CT with contrast and PET-CT. In patients with metastatic disease or a high risk of metastases, assessment of HLA-A*02:01 is recommended for potential treatment with tebentafusp [27,28]. Additional lab tests, such as liver function, renal function, hematology, and LDH (lactate dehydrogenase), should also be performed. Patients with elevated LDH usually have more advanced disease and a worse prognosis [29]. The assessment of ctDNA at the metastatic setting diagnosis and throughout therapy to monitor disease activity is also recommended [30].

## 3. Treatment of Metastatic Uveal Melanoma

**Figure 2:**
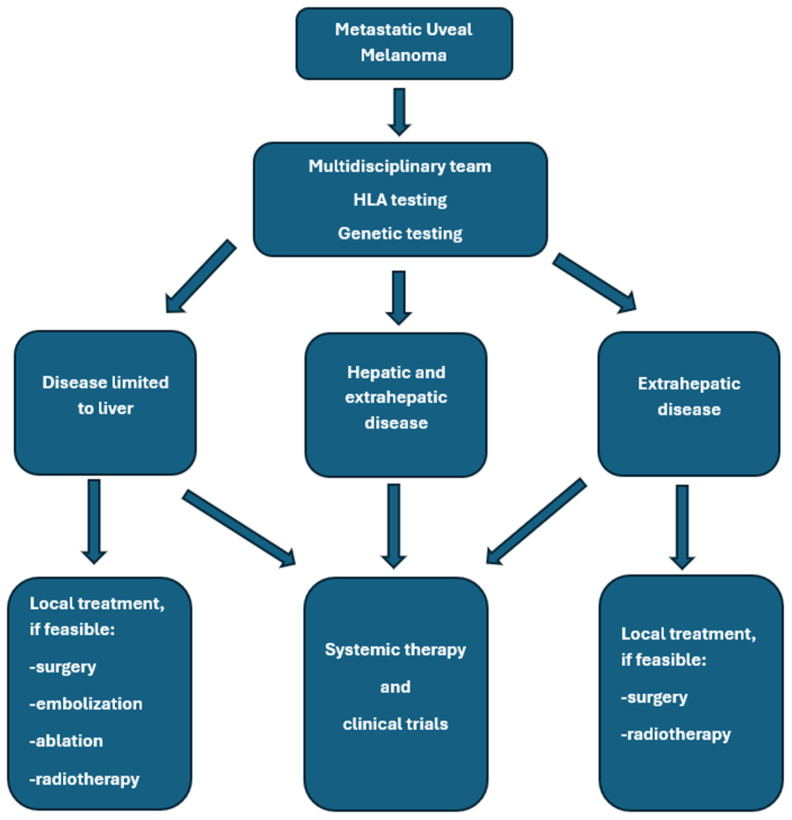
General rules of metastatic uveal melanoma management.

Treatment of mUM requires a multimodal approach, which may include local and systemic therapies. Liver-directed therapies remain an essential component in the management of hepatic metastases of UM and can provide effective local control, symptomatic relief, and potential survival benefits in carefully selected patients [20,31,32]. Systemic treatment options are limited in efficacy [31]. Khoja et al. published the results of a meta-analysis based on data from 965 patients with mUM who participated in 29 prospective studies. The median progression-free survival (mPFS) was 3.3 months in the entire group of patients; the PFS was 5.2 months in the liver-directed therapy group, 2.8 months in patients treated with immunotherapy, kinase inhibitors, or anti-angiogenic drugs, and 2.6 months in the chemotherapy group. The median OS (median OS) was 10.2 months in all enrolled patients and reached 14.6 months in the liver-directed therapy-treated group, and, respectively, 11.0, 9.2, 9.1, and 8.9 months for the groups treated with anti-angiogenic drugs, chemotherapy, kinase inhibitors, and immunotherapy. Univariate analysis revealed that shorter PFS was associated with liver metastases greater than 3 cm, elevated lactate dehydrogenase, and elevated alkaline phosphatase [33]. Personalized treatment planning based on tumor burden and location, disease course, hepatic function, patient performance status, comorbidities, and their treatment, HLA-A*02:01 genotype testing, is essential for the proper treatment of patients with mUM. A valuable option remains participation in clinical trials if available [32]. In general, surgery provides superior local control and OS improvement compared to systemic therapy alone. Hepatic metastasectomy or partial hepatectomy remains a valuable option for patients with limited disease. Only selected patients with liver metastases from UM are good candidates for complete metastasis resection, mainly due to the spread of multifocal malignancy. This treatment should be used in patients with an expected long survival time, without extra-hepatic metastases, and with potentially radically resected lesions (R0). Usually, two liver segments are removed. Retrospective analyses suggest that complete resection may improve OS, especially if complete resection is feasible. In different studies, the complete resection rates ranged from 27% to 88% [34,35,36]. For example, Mariani et al. reported 30% R0 resections in a group of 255 patients treated at a single center in France [37]. In this study, mOS in all patients treated with surgery, irrespective of the completeness of resection, was 14 months compared to 8 months in patients who did not undergo surgery, and was 27, 17, and 11 months after complete surgical resection (R0), R1 resection, and R2 resection, respectively. Three percent of patients died within 30 days after surgery due to liver insufficiency, hemorrhagic events, and subphrenic abscess. Based on a systematic review of 793 patients, OS ranged from 10 to 35 months in patients treated with surgery [38]. The general summary of treatment options is shown in Figure 2 and in Table 2.

## 4. Systemic Treatment in Metastatic Uveal Melanoma

Metastatic uveal melanoma (mUM) is characterized by poor prognosis and limited systemic treatment options [55,56]. Multiple clinical trials have not shown any benefit from the use of the tested therapeutic options. The first approved systemic therapy with OS benefit in patients with HLAA*02:01-positive nonresectable mUM is tebentafusp. New molecules are being developed in clinical studies, and participation in these trials is strongly recommended unless local therapies are more suitable [56,57].

In view of the historically limited efficacy and unfavorable toxicity profile of conventional cytotoxic chemotherapy, contemporary clinical practice guidelines and recent systematic reviews increasingly emphasize a transition toward precision-based approaches, particularly targeted agents and immuno-oncologic therapies [21,28,58,59]. Nevertheless, systemic chemotherapy can retain clinical utility in highly selected patient populations, most notably in settings where approved targeted or immune-based strategies are unavailable, contraindicated, or accessible exclusively within clinical trial frameworks. Consequently, chemotherapy is mainly relegated to a palliative role. In contrast, current standards of care prioritize enrollment in well-designed clinical trials and the implementation of evidence-based targeted or immunotherapeutic regimens when appropriate. Crucially, comprehensive multidisciplinary evaluation at specialized referral centers remains indispensable to optimizing individualized treatment strategies and ensuring equitable access to emerging therapeutic modalities within this rapidly evolving landscape [20,21,58,59,60].

### 4.1. Chemotherapy

Systemic chemotherapy generally shows minimal efficacy in mUM. Several chemotherapy regimens have been evaluated in prospective trials in this patient population, but it remains unclear whether monotherapy improves survival. Single-agent treatments, including dacarbazine, fotemustine, and temozolomide, as well as combinations such as treosulfan plus gemcitabine, have demonstrated low ORR, with mPFS typically under 4 months, and mOS around 10 months [60,61,62]. Standard first-line triplet chemotherapy with cisplatin, vinblastine, and dacarbazine yielded an ORR of 20%, mOS of 13 months, and PFS of 5.5 months [63]. In a phase 2 trial, treosulfan combined with gemcitabine achieved an ORR of 28.6%, mOS of approximately 14 months, PFS of about 6.5 months, and 1-year survival of 80% [64]. Across four prospective clinical trials evaluating combination chemotherapy protocols, median progression-free survival ranged from 2.5 to 6.7 months, while median overall survival varied between 7.5 and 14.2 months [60,61,62]. Additionally, clinical trial data indicate therapeutic activity of the BOLD regimen—comprising dacarbazine, vincristine, bleomycin, and lomustine—when administered in combination with interferon [65]. Other studies confirm that objective responses are rare, and survival benefits are marginal, underscoring the substantial chemoresistance of UM [66,67].

### 4.2. Immunotherapy

Immune checkpoint inhibitors (ICIs), targeting PD-1 (nivolumab, pembrolizumab) and CTLA-4 (ipilimumab), have been evaluated in multiple clinical trials and real-world studies; however, their efficacy is generally lower than in cutaneous melanoma [56,58,68]. Meta-analyses and clinical trials have shown that the treatment results are not superior to conventional chemotherapy. Single-agent anti-PD-1 therapy in mUM showed low ORR (3–5%), with mPFS of 2–3 months and mOS of 7–10 months [69,70]. Ipilimumab monotherapy yields similar ORR (0–10%) and OS (6–10 months), with grade ≥ 3 immune-related adverse events in up to 36% of patients [70,71,72]. Combination therapy with ipilimumab plus nivolumab increases ORR to 11–17%, mPFS to 3–4 months, mOS 12.7–18 months, but grade ≥ 3 toxicity rises to 40–54%, predominantly colitis, hepatitis, and endocrinopathies [36,55,73]. A combination of anti-PD1 and anti-LAG3 monoclonal antibodies has been assessed in a phase 2 study (NCT04552223, n = 27). The safety profile was consistent with previous data, but the ORR was low (7.7%) [74]. Nevertheless, combination nivolumab of with relatlimab as salvage treatment has been reported as successful in selected cases [75].

Retrospective analyses confirmed the limited activity of immunotherapy in mUM. To compare the efficacy of chemotherapy used before 2017 and immunotherapy used after 2017, Magomedova et al. analyzed data from 124 patients treated with chemotherapy and 144 treated with immunotherapy. The chemotherapy regimens used the most frequently were cyclophosphamide plus vincristine plus dacarbazine (n = 39) and paclitaxel plus carboplatin (n = 18). In the immunotherapy group, patients were treated with anti-PD-1 (n = 78), ipilimumab plus nivolumab (n = 48), ipilimumab (n = 10), and tebentafusp (n = 8). In the immunotherapy group, 26 patients received additional liver-directed therapies. Grade 3 and 4 treatment-related AEs were observed in 28.2% and in 30.6% of patients in the chemotherapy group and immunotherapy group, respectively. The survival was prolonged with immunotherapy in comparison to chemotherapy, mPFS was 4.1 months vs. 1.8 months and mOS 21.7 months vs. 5.6 months. There were no responses in the chemotherapy group while 11.8% of patients treated with immunotherapy achieved response (4-CR, 13-PR) [76].

### 4.3. Tebentafusp

**Figure 3:**
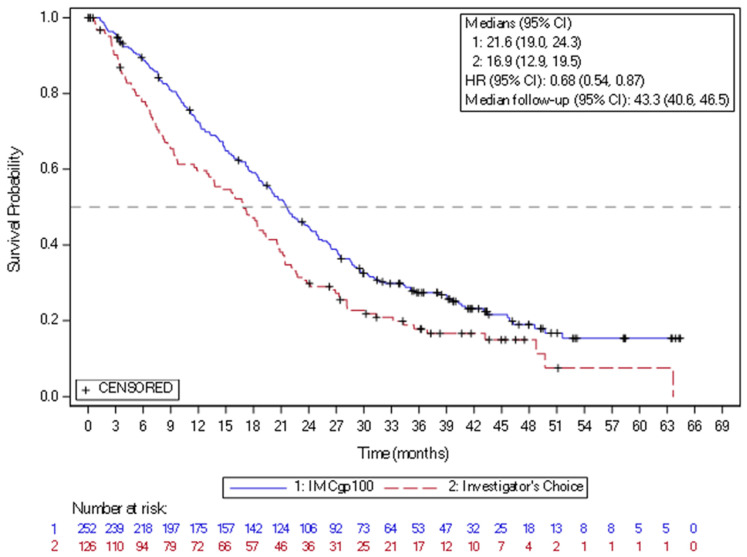
Kaplan–Meier curves of overall survival in the study of IMCgp100-202 (3-year follow-up analysis) in the intention-to-treat population. CI = confidence interval; HR = hazard ratio; IMCgp100 = tebentafusp; ITT = Intent-to-treat.

In 2022, the new drug, tebentafusp, a bispecific gp100 peptide-HLA-directed CD3 T-cell engager, had been approved by FDA and EMA, based on the positive efficacy results in mUM. Tebentafusp is a bispecific fusion protein composed of a T-cell receptor (TCR)-targeting moiety linked to an anti-CD3 antibody fragment that serves as the effector component. The TCR domain exhibits high-affinity binding to a gp100 peptide presented by HLA-A*02:01 on the surface of UM cells, while the effector domain engages the CD3 receptor in polyclonal T cells. This drug demonstrated a reproducible OS benefit in prospective trials and real-world series. In the randomized phase 3 IMCgp100-202 study (NCT03070392) (n = 378), the patients with previously untreated HLA-A02:01-positive mUM were randomized to tebentafusp arm or control group treated based on investigator choice with pembrolizumab, ipilimumab, or dacarbazine. The mOS was 21.6 months in the tebentafusp group and 16.9 months in the control group (HR 0.68) (Figure 3). The PFS rate at 6 months was 31% vs. 19, despite modest ORR around 9%. The most frequently observed treatment-related adverse events of any grade in the tebentafusp group were rash (83%), pyrexia (76%), pruritus (70%), and hypotension (38%). Grade 3 or 4 treatment-related adverse events occurred in 109 patients (44%) in the tebentafusp group compared with 19 patients (17%) in the control group. Extended follow-up confirmed a durable survival benefit, with a 3-year OS of 27% vs. 18%. No new safety signals were observed [77]. Tebentafusp is indicated as monotherapy for the treatment of human leukocyte antigen (HLA)-A*02:01-positive adult patients with unresectable or mUM. This medication is given weekly, with the dose increased for the first three infusions and close patient follow-up lasting at least 16 h after the first three infusions, mostly due to the risk of cytokine release syndrome. Based on the data from two clinical trials with tebentafusp in mUM (IMCgp100-102 and IMCgp100-202, n = 378), the most frequently reported AEs in patients receiving tebentafusp included cytokine release syndrome (88%), rash (85%), pyrexia (79%), pruritus (72%), fatigue (66%), nausea (56%), chills (55%), abdominal pain (49%), edema (49%), hypo- or hyperpigmentation (48%), hypotension (43%), dry skin (35%), vomiting (34%), and headache (32%). Four percent of patients required treatment discontinuation due to AEs [19,27].

Real-world evidence confirmed the efficacy of tebentafusp in mUM. A multicenter real-world cohort study including centers in Belgium, France, and Poland confirmed these findings, showing tebentafusp to be effective and safe in a broad patient population (n = 175), with high-risk features such as elevated LDH and extrahepatic metastases correlating with shorter OS. mPFS was 4 months (95% CI 2.7–5.3), and mOS was 20 months (95% CI 15.3–24.6) with 1-year OS rate of 63.6% [78]. In the Department of Soft Tissue/Bone Sarcoma and Melanoma Maria Sklodowska-Curie National Research Institute of Oncology in Poland, over 550 UM patients at different stages have been treated and followed. Among them, 132 patients were treated with chemotherapy, a few with immunotherapy, and 50 with tebentafusp. The most frequently used chemotherapy regimens were CVD (63 patients) and BOLD (47 patients). mOS in patients treated with chemotherapy containing dacarbazine was 9.0 (7.2–11.4) months [n = 98] vs. 13.4 months (n = 32) in patients treated with tebentafusp. Notably, approximately 50% of patients treated with tebentafusp continued treatment beyond disease progression. Tebentafusp safety profile was consistent with that previously reported [79].

Based on the reported analyses, early reductions in ctDNA after tebentafusp treatment may be associated with improved OS in patients with metastatic uveal melanoma, which was shown in the clinical trial. Patients with early ctDNA reduction had prolonged mOS compared to patients with lower reduction; the mOS was 21.2 months vs. 12.9 months. Although retrospective and early-phase analyses suggest this correlation, prospective validation is still required. In addition, patients with detectable ctDNA before starting treatment with tebentafusp had significantly lower mOS (12.9 months vs. 40.5 months) and mPFS (2.5 months vs. 10.8 months) [30]. In summary, tebentafusp consistently provides a clinically meaningful OS benefit across randomized and observational datasets, regardless of RECIST (Response Evaluation Criteria in Solid Tumors) responses or conventional PFS.

J.M. Piulats et al. compared OS on tebentafusp or pembrolizumab in the IMCgp100-202 study with nivolumab plus ipilimumab in the GEM1402 study in previously untreated patients with mUM using the inverse probability of treatment weighting based on the propensity score (IPTW) [80]. The IPTW-adjusted analysis demonstrated that tebentafusp significantly favored OS (HR 0.52; 1-year OS was 73% for tebentafusp vs. 50% for nivolumab plus ipilimumab). Sensitivity analyses confirmed the superior OS for tebentafusp. The findings support the conclusion that tebentafusp provides a similar OS benefit compared with nivolumab plus ipilimumab.

### 4.4. Targeted Therapy

Metastatic UM is characterized by distinct genetic alterations compared to cutaneous melanoma, notably activating mutations in *GNAQ* and *GNA11*, and secondary alterations in *BAP1*, *SF3B1*, and *EIF1AX* [81]. These molecular insights have guided the development of targeted therapies, including MEK inhibitors, PKC inhibitors, and other pathway-directed agents. In most studies with targeted therapy, the ORR was up to 10% [82,83]. UM often express KIT, but the *c-KIT* mutations are of low frequency and imatinib showed limited efficacy in clinical trials. In a phase 2 study with 25 patients with mUM enrolled and treated with imatinib, mPFS was 2.8 months and mOS was 6.9 months [84].

Selumetinib, an MEK1/2 inhibitor, was evaluated in a randomized phase 2 trial against chemotherapy (temozolomide or dacarbazine) in patients with mUM (NCT01143402). A modest improvement in PFS, mPFS of 15.9 vs. 7 weeks and ORR of 14% vs. 0% was reported, and there was no improvement in OS, mOS, which was 11.8 vs. 9.1 months [85]. Generally, the targeted therapy in biomarker-unselected patients is of limited benefit in mUM [86].

Promising results were observed in a phase 2 trial using molecularly targeted therapy, using crizotinib in combination with the protein kinase C (PKC) inhibitor darovasertinib, which targets *GNAQ/GNA11* mutations, in patients with mUM (NCT03947385). In this study, 30% of patients achieved a response to treatment, and another 60% achieved stable disease [87,88].

### 4.5. Other Systemic Treatments for Metastatic Uveal Melanoma

Antibody–drug conjugates, which are widely used in some malignancies, have been tested in early-phase clinical trials in patients with melanoma, including uveal melanoma. Glembatumumab vedotin, a fully human IgG2 monoclonal antibody directed against the glycoprotein NMB coupled by a peptide linker to monomethyl auristatin E, was evaluated in the phase 2 clinical study (NCT02363283) in patients with mUM or locally recurrent UM. The ORR was 6%, and DCR 57%, with mPFS of 3.1 months and OS 11.9 months [89]. In a phase 1 study of the anti-endothelin B receptor antibody–drug conjugate DEDN6526A in patients with metastatic or unresectable cutaneous, mucosal, or UM (NCT01522664), DEDN6526A at the selected dose showed an acceptable profile and preliminary antitumor activity in all melanomas [90].

Treatment of mUM with adoptive transfer of tumor-infiltrating lymphocytes has also been assessed in clinical trials. In the single-center phase 2 study (NCT01814046), 21 patients with UM were enrolled. Autologous TILs cultures were generated after metastasectomy, and patients received lymphodepleting conditioning chemotherapy before infusion of autologous TILs and high-dose interleukin-2. Eight patients responded to treatment. The AEs were mostly related to lymphodepleting chemotherapy [91].

The use of oncolytic viral therapy has been assessed in clinical trials in patients with mUM. RP2, a selective, replication-competent HSV-1 engineered to express GM-CSF, a modified GALV-GP glycoprotein, and an anti-CTLA-4 antibody-like molecule, has been evaluated in a phase 1 study (NCT04336241) in patients with mUM. In total, 17 patients received treatment either in monotherapy (n = 3) or in combination with nivolumab (n = 14). The ORR was 29.4%, and DCR 58.8%. The most common AEs were pyrexia, chills, fatigue, hypotension, and pruritus [92]. The data about cancer vaccine use in mUM are limited. Bol et al. reported responses induced with dendritic cell vaccination in four (29%) of 14 patients, with mOS of 19.2 months and no severe treatment-related toxicities [93].

In the phase 1a/1b study (NCT03686124), 16 adult HLA-A*02:01+ patients with UM were treated with IMA203, a PRAME-directed TCR T-cell therapy engineered to recognize intracellular PRAME-derived peptides presented by HLA. Following leukapheresis and the manufacture of IMA203, the patients underwent lymphodepletion and IMA203 infusion followed by low-dose IL-2. The median of previous lines of therapy was two (62.5% prior tebentafusp), 75% patients had the largest metastatic lesion measuring 3.1–8.0 cm in diameter, and 56% had elevated LDH. ORR was 67%; tumor shrinkage was observed in all participants. Median DOR was 11.0 months, and mPFS and mOS were 8.5 months and 16.2 months, respectively. Tolerability was favorable [94,95].

Recent studies show benefit from a precision oncology approach based on molecular testing and tumor mutational burden testing [86,96].

## 5. Local Therapies in Metastatic Uveal Melanoma

The results of local treatment have been confirmed in many prospective analyses and in a systematic review published in 2025 [16,97]. Surgery, described previously in Section 3, is a valuable option in eligible patients. Radiofrequency ablation (RFA), microwave ablation, and cryoablation are minimally invasive options for small lesions (<3 cm). Local control rates exceed 70–80% for appropriately sized tumors, and procedure-related morbidity is generally low [52,97,98]. Various techniques have been developed for the localized delivery of pharmaceutical therapies to the liver, allowing the administration of higher doses than would be tolerable systemically due to toxicity constraints. These techniques include embolization-based therapies, percutaneous hepatic perfusion (PHP), and isolated hepatic perfusion (IHP). Transarterial therapies, including transarterial chemoembolization (TACE) and transarterial radioembolization (TARE, e.g., Yttrium-90) are also valuable options in patients with UM with liver metastases.

### 5.1. Therapies Based on Liver Metastases-Directed Embolization

Isolated hepatic perfusion—IHP—and percutaneous hepatic perfusion—PHP—enable perfusion of the liver with high-dose chemotherapy while minimizing systemic exposure. Isolated hepatic perfusion (IHP) is a more prolonged and complex procedure than PHP and is used infrequently. It is an open surgical procedure and requires vascular isolation of the liver to allow the delivery of high doses of heated chemotherapy, for example, melphalan or cisplatin, directly to the organ through an arterial catheter. It requires a few days of hospitalization and can cause significant complications, including transient hepatotoxicity and portal vein thrombosis [18,42].

Prospective studies of PHP with melphalan report hepatic response rates of 30–40% and mOS up to 16 months [99,100,101,102]. A phase III randomized trial of hepatic intra-arterial fotemustine vs. IV fotemustine (ClinicalTrials.gov NCT00110123) showed no OS benefit (14.6 vs. 13.8 months) but improved PFS (4.5 vs. 3.5 months) and RR (10.5% vs. 2.4%) [103]. Nevertheless, based on the FOCUS phase 3 trial (NCT02678572), PHP with melphalan had been approved by the FDA for UM with liver metastases. In this study, patients were treated with PHP with melphalan or the investigator’s choice of TACE, pembrolizumab, ipilimumab, or dacarbazine. Patients treated with PHP with melphalan had an ORR (objective response rate) of 35.2% (n = 91) compared to 12.5% in the comparator group (n = 42), with a median duration of response of 14 months, and mOS of 20.5 months compared to 14.1 months in the control group. Adverse events were observed in 42.6% of patients in the group receiving liver-directed treatment with melphalan, but these were mostly transient. No death had been reported in the treatment group [104]. PHP is associated with hematologic toxicity, primarily thrombocytopenia and neutropenia, which are usually reversible [17].

In the SCANDIUM phase 3 clinical trial (NCT01785316), 87 patients with UM with previously untreated isolated liver metastases were randomized in a 1:1 ratio to the group treated with melphalan IHP (n = 41) or to the control group treated according to the choice of the investigator (chemotherapy, immunotherapy, or other localized interventions) (n = 44). The mOS was 21.7 in the IHP group vs. 17.6 months in the control group (HR: 0.64; 95% CI: 0.37–1.10) and mPFS was 7.4 months in the IHP group vs. 3.3 months in the control group (HR = 0.21, 95% CI, 0.12 to 0.36) [100,105].

### 5.2. Transarterial Therapies Directed to Liver Metastases

Transarterial chemoembolization (TACE) is a liver-directed catheter-based therapy that combines hepatic-arterial chemotherapy with embolization. TACE results in response rates of 20–35% and an mOS of around 10–14 months [16]. In a first TACE clinical trial using cisplatin (±carboplatin) followed by polyvinyl alcohol embolization, partial responses were reported in 57% of patients with median time to progression of 8.5 months and median survival 11.5 months after first TACE (17 vs. 11 months when liver tumor burden was <25% vs. >25%) [51]. Subsequently, in a larger series, chemoembolization yielded a partial response rate of 11%, along with mPFS of 3.8 months, and mOS 6.7 of months [106]. At the same time, in an Asian cohort treated with cisplatin (and gelatin sponge), ORR was 21%, with mOS reaching 23 months, and 1-, 2-, and 5-year survival rates of 72.4%, 39.4%, and 0%, respectively. In these patients, liver enzyme elevation was reported in 100% of them, with grade ≥ 3 AST/ALT elevations of 34.5% and 51.7%, respectively [48]. A small prospective first-line study using irinotecan drug-eluting beads reported an ORR of 80%, with all patients alive at ~10–16 months of follow-up [47]. TACE is often repeated and may be used alone or alongside systemic therapy. The efficacy of tebentafusp + TACE has not been reported yet, but transarterial immunoembolization with concurrent checkpoint blockade was reported to induce an ORR of 17%, mPFS of 4.9 months, and mOS of 35 months, at the price of immune-related hepatitis during such combination therapy [107].

Transarterial radioembolization—TARE, also termed selective internal radiation therapy (SIRT)—delivers intra-arterial Yttrium-90 (90Y) microspheres (made of resin or glass) via catheter into the hepatic artery to locally irradiate uveal melanoma metastases while sparing normal liver parenchyma. TARE is typically performed as a lobar or segmental procedure after angiographic mapping. It is used either as a standalone liver metastases-directed therapy or with systemic therapy [108]. TARE uses beta radiation. It was demonstrated to induce disease control rates of 60–70% and mOS of 12–15 months in several retrospective series [98]. In the prospective phase II radiology trial, reported ORR was 18%, with hepatic mPFS of 5.5 months, and mOS of 19.1 months resulting from 1-year OS as high as 70.4% [109]. When used in a first-line treatment, SIRT induced partial responses in 30% of cases and disease control of 80%, with mPFS of 8.1 months and mOS of 18.7 months [45].

A systematic review of SIRT for uveal melanoma liver metastases reported median OS of 12.3 months and hepatic PFS of 5.4 months across included studies [46]. German multicenter registry analysis confirmed longer OS with liver-directed therapy plus immune checkpoint blockade vs. immune checkpoint blockade only (20.1 vs. 13.8 months) and a higher ORR for multidisciplinary treatment compared to immune checkpoint blockade (16.7% vs. 3.8%) [110]. Moreover, sequential SIRT and nivolumab with ipilimumab was associated with grade 3–4 immune-related adverse events in 44.4% and an mOS from metastatic diagnosis of 49.6 months [111]. At this point in time, tebentafusp–TARE combinations are being prospectively evaluated (e.g., NCT06627244), but efficacy results are not yet available.

For both TACE and TARE, post-embolization syndrome, transient elevations in liver enzymes, and fatigue are common [47,50,112].

### 5.3. Liver-Directed Radiotherapy

Stereotactic body radiation therapy (SBRT) may be a valuable option in case other liver-directed therapies are not feasible and for patients with extrahepatic metastases. SBRT provides high-dose, precisely targeted radiation to hepatic lesions while sparing surrounding liver tissue. EBRT, though less precise, can be used in palliative settings to reduce tumor burden and alleviate symptoms (Table 2) [20,66,68].

### 5.4. Local Treatment of Extrahepatic Disease

Isolated extrahepatic metastases are rare in UM. Extrahepatic metastases in UM are most commonly located in the lung, bone, and central nervous system, often in the context of multiorgan metastatic disease. In a multicenter cohort of 1845 metastatic UM patients, symptomatic brain metastases were identified in 6.3%, and median survival after brain metastases diagnosis was 7.6 months [113]. The data about local treatment of extrahepatic metastases is limited and this treatment is not considered the standard of care. In highly selected cases with isolated or oligometastatic disease, absence of hepatic metastases, long disease-free intervals, surgery, and radiotherapy including radiosurgery may be considered based on a multidisciplinary team (MDT) decision. Management is typically individualized per other solid-tumor oligometastatic practices including surgical metastasectomy for technically resectable solitary lesions, and stereotactic or conventionally fractionated radiotherapy for CNS, bone or spine metastases, and selected thoracic metastases. Systemic therapy remains essential because extrahepatic lesions frequently coexist with occult micrometastatic liver disease [113,114,115].

## 6. Liver-Directed Therapy in Combination with Systemic Treatment

Integration of liver-directed therapies with systemic approaches, including ICI and molecularly targeted agents, is an area of growing clinical and translational interest. Emerging evidence indicates that achieving local tumor control may potentiate systemic immune activation, thereby augmenting the therapeutic efficacy of immunotherapy, particularly in patients with liver-dominant disease [98,101,116].

The evolving therapeutic strategy for mUM with predominant hepatic involvement focuses on the integration of liver-directed locoregional modalities with systemic immunotherapies. Retrospective data from a single-center cohort (n = 45) indicate that the combination of ICI and liver-directed therapies (e.g., surgical resection or transarterial approaches) significantly prolongs OS—mOS of 22.5 months vs. 11.4 months with systemic therapy alone [117]. Furthermore, a larger retrospective multicenter analysis reveals that patients receiving liver-directed therapy as first-line treatment demonstrated a median melanoma-specific survival of 28 months vs. 10 months for systemic therapy alone, with further survival gains observed when followed by ICI [118].

In prospective early-phase studies, sequential application of selective internal radiation therapy (SIRT) using Yttrium-90 followed by combination ICI (ipilimumab + nivolumab) yielded overall response rates and survival outcomes that support a synergistic interaction, despite the need for dose modifications due to toxicity [111]. These findings are echoed in additional retrospective investigations, which affirm a more favorable mOS when systemic ICI is combined with liver-directed interventions compared to either modality alone [118].

In the SCANDIUM II phase 1b study (NCT01785316), IHP plus systemic treatment with nivolumab and ipilimumab was compared with immunotherapy alone. The number of patients enrolled was small (N = 18). However, based on preliminary results, it appears that the combination of IHP with immunotherapy may be feasible in patients with liver-dominant metastatic UM [119].

In the CHOPIN phase 2 study (NCT04283890) (n = 79), the addition of ipilimumab and nivolumab to PHP in mUM patients with hepatic-only or hepatic-dominant disease significantly improved PFS (12.8 vs. 8.3 months), OS (23.1 vs. 19.6 months), and ORR (76.3% vs. 39.5%) with a manageable safety profile [43,120].

## 7. Drugs in Development

Multiple agents are currently in development, targeting key oncogenic pathways and the immune microenvironment to improve outcomes. The current pipeline includes antibody-based therapies, adoptive cell transfers, combination regimens of targeted agents, and novel immunomodulatory strategies to improve efficacy and overcome intrinsic resistance mechanisms [32]. Recent advances in genomic, transcriptomic, and broader multi-omic profiling have provided a more comprehensive understanding of tumor biology, facilitating biomarker-driven patient stratification and the identification of actionable molecular targets [83]. Integrating these multidimensional datasets with clinical outcomes has allowed the design of rational combination therapies and predictive frameworks to anticipate the response to treatment. Continued translational efforts connecting molecular insights with therapeutic development are essential to advance personalized interventions. In addition to strategies for advanced disease, ongoing studies also focus on interventions to reduce local recurrence and metastatic spread in high-risk patients (Table 3).

## 8. Discussion

This article provides a comprehensive overview of the current standards of care for UM, highlighting significant advances in diagnostics and therapy while also pointing out persistent challenges, particularly in the context of metastatic disease. Unlike cutaneous melanoma, UM is characterized by a unique biology and a predilection for liver metastases, which is a key factor influencing prognosis.

The prognosis for metastatic UM (mUM) remains poor, with an mOS ranging from 3 to 30 months, which is mostly due to the limited efficacy of systemic therapies. Considering this, liver-directed therapies, such as surgical resection, embolization techniques (TACE, TARE), ablative and other methods like PHP and IHP, and SBRT, have become the cornerstone of treatment in patients with disease limited to the liver and eligible for local treatment. These therapies, particularly complete resection, have been shown to significantly improve median survival compared to systemic treatment. Notably, data from the phase 3 FOCUS trial confirms that PHP with melphalan provides better ORR and OS compared to conventional therapies. The isolated extrahepatic disease is rare and is associated with better prognosis, and despite data about local treatment being limited, the local therapies, including surgery and radiotherapy, may be considered in selected cases based on the MDT decision.

The efficacy of conventional chemotherapy in mUM is generally limited. Similarly, ICI therapy shows limited activity compared to cutaneous melanoma, although “real-world” data suggest a small benefit, especially in combination with liver-directed therapies. A breakthrough is the approval of tebentafusp, the first systemic agent to demonstrate a significant advantage in OS in the IMCgp100-202 study, which has been confirmed in daily practice. Importantly, early reduction in ctDNA after tebentafusp treatment may be an early indicator of a better prognosis, even in cases of radiologically assessed disease progression, suggesting the need for further research on biomarkers.

The emerging opportunities, including systemic treatment for HLA-A*02:01-negative patients, a combination of local treatment with systemic therapies leading to further survival improvement, and development of new drugs targeting known molecular abnormalities based on multi-omic profiling studies, may change the treatment paradigm in the near future. A key factor in optimizing treatment is an individualized approach to the patient, considering the molecular profile of the tumor (e.g., *BAP1*, *SF3B1*, *EIF1AX*, *PRAME* mutations) and HLA-A*02:01 status, as well as participation in clinical trials. The proper management should be personalized and include MDT discussion, especially in candidates for local treatment, local treatment combined with systemic therapy, and patients with extrahepatic disease only.

## 9. Conclusions

UM is a rare but aggressive cancer with a high risk of metastases, particularly to the liver, despite great opportunities for primary tumor treatment. In spite of the limited efficacy of systemic therapies, there is some progress observed in the treatment of patients with mUM. Liver-directed strategies remain the cornerstone of therapy, offering the most favorable outcomes for liver metastases. New systemic approaches, such as tebentafusp, represent a promising advance, providing significant OS benefit for a selected group of patients. Future research should focus on further understanding the genetic and molecular underpinnings of UM, as well as on identifying new systemic therapies, including drug combinations that can overcome chemoresistance and induce lasting immune responses. The implementation of individualized treatment plans in specialized centers within MDT, supported by precise molecular diagnostics, availability of local and systemic treatments, and access to clinical trials, is crucial for improving treatment outcomes and the prognosis for patients with mUM.

## Figures and Tables

**Table 1:** Follow-up after primary uveal melanoma treatment—summary of recommendations [20,21,22,23].

Risk Group	Characteristics of a Risk Group	Recommendations
Patients with ocular melanoma at low risk for distant metastases	Stage T1 * and known molecular abnormalities (disomy of chromosome 3, multiple copies of 6p, *EIF1AX* mutation)	imaging tests if indicated
Patients with ocular melanoma at intermediated risk of distant metastases	Stage T2 * or T3 * or with known molecular abnormalities (*SF3B1* mutation)	Imaging tests every 6–12 months and if clinically indicated
Patients with ocular melanoma at high risk for distant metastases	Stage T4 * or with known molecular abnormalities (monosomy of chromosome 3, multiple copies of 8q, *BAP1* mutation, PRAME expression)	Follow-up imaging tests every 3 to 6 months for 5 years, then every 6 to 12 months for up to 10 years, then as clinically indicated (signs or symptoms)

* According to AJCC, the primary ciliary body and choroidal melanomas (T, tumor) are classified according to the four tumor size categories from T1 to T4 based on the thickness and the largest basal diameter in mm.

**Table 2:** Treatment options in metastatic uveal melanoma.

Treatment Mode	Drug	Indications	Schedule	Endpoint	Adverse Events	Reference
Metastatic (systemic)	Tebentafusp (ImmTAC; HLA-A*02:01+)	1L preferred in HLA-A*02:01+ non-resectable/metastatic UM	Weekly IV with step-up dosing	Overall randomized—survival advantage sustained at ≥3 years; Real effectiveness—world confirmed	Cytokine-mediated AEs (fever, rash, hypotension) early; manageable with protocols	[19]
Metastatic (systemic)	Dual immune checkpoint blockade (nivolumab + ipilimumab)	HLA-A*02:01− or post-tebentafusp; fit PS; consider liver-dominant vs. extrahepatic disease	Standard melanoma schedules	Disease control in subset; response rates lower than in cutaneous melanoma	Immune-related AEs; careful selection and monitoring	[39,40,41]
Metastatic (liver-directed)	Percutaneous hepatic perfusion (chemotherapy)	Unresectable liver metastases-dominant; limited extrahepatic burden	Repeatable M-PHP every 6–8 weeks (up to ~6 cycles)	Objective responses around one-third; median PFS ~9 months; median OS ~20 months in the pivotal cohort	Transient myelosuppression; Short-term QoL dip with recovery within weeks	[17,42,43]
Metastatic (liver-directed)	Radioembolization (Y-90)	Liver-predominant disease; segmental/lobar distribution	Center-specific activity planning; usually single session(s)	Disease control frequent in retrospective series; prospective comparative data limited	Post-embolization fatigue; rare radiation-induced liver disease	[44,45,46]
Metastatic (liver-directed)	Transarterial (chemo) embolization (TACE/TAE)	Multifocal hepatic disease; when M-PHP/radioembolization unsuitable	Drug-eluting beads or bland embolization protocols	attainable disease control; survival varies with burden and sequencing	Post-embolization syndrome, LFT flares; repeatable	[47,48,49,50,51]
Metastatic (surgical/ablative)	Resection/Ablation (RFA/MWA)	Oligometastatic liver ± limited extrahepatic disease; favorable anatomy	Anatomical /atypical resection; image-guided thermal ablation	Long-term survivors were selected; best outcomes with low tumor number/volume	Procedure risks; requires MDT and careful imaging follow-up	[52,53,54]

Abbreviations: AE, adverse event; M-PHP, melphalan percutaneous hepatic perfusion; PS, performance status; Y-90, Yttrium-90; TACE, transarterial chemoembolization; TAE, transarterial embolization; RFA, radiofrequency ablation; MWA, microwave ablation; HLA-A*02:01, human leukocyte antigen A*02:01; PFS, progression-free survival; OS, overall survival; QoL, Quality of life: LFT, liver function test; MDT, multidisciplinary team.

**Table 3:** Summary of clinical trials investigating systemic treatments for metastatic uveal melanoma (based on https://clinicaltrials.gov, access date 20 August 2025).

**Targeted therapy in perioperative setting**
Phase 2 study of (neo)adjuvant IDE196 (darovasertib) in patients with localized ocular melanoma (NCT05907954)
**Immunotherapy in an adjuvant setting**
Phase 3 study of adjuvant tebentafusp in high-risk ocular melanoma (ATOM) (NCT06246149)
**Local treatment combined with systemic therapy**
Phase 2 study of neoadjuvant tebentafusp in patients with mUM with resectable/potentially resectable liver metastasis and absence of extrahepatic disease (NCT07057596)
Phase 2 study of concurrent SBRT with niwolumab and relatlimab in mUM (NCT05077280)
Phase 2 study of tebentafusp and trans-arterial radioembolization with Yttrium-90 in the treatment of mUM (NCT06627244)
Phase 3 study of PHP in combination with IPI1/NIVO3 compared to IPI3/NIVO1 only in patients with liver mUM (NCT06519266)
**Immunotherapy alone or in combination with other systemic treatment in an advanced/metastatic setting**
Phase 2 study of tebentafusp in HLA-A*0201-positive previously untreated mUM with an integrated ctDNA biomarker (NCT06070012)
Phase 1/2 study of different doses of BI-1607 in combination with pembrolizumab and ipilimumab, in participants with unresectable mUM (NCT06784648)
Phase 2 study of roginolisib in patients with advanced/mUM (NCT06717126)
Phase 2/3 study to investigate the efficacy and safety of RP2 in combination with nivolumab in immune checkpoint inhibitor-naïve adult patients with mUM (NCT06581406)
Phase 2 Study of olaparib in combination with pembrolizumab for advanced UM (NCT05524935)
Phase 2/3 study of IDE196 (darovasertib) in combination with crizotinib as first-line therapy in mUM (NCT05987332)
Phase 2 study of cemiplimab plus ziv-aflibercept for subjects with mUM (NCT06121180)
**Targeted therapy in an advanced/metastatic setting**
Phase 2 study of binimetinib plus belinostat for subjects with mUM NCT05170334

## Data Availability

No new data was created or analyzed in this study.

## Abbreviations

The following abbreviations are used in this manuscript:

AJCC	American Joint Committee on Cancer
BAP1	BRCA1-associated protein 1
COMS	Collaborative Ocular Melanoma Study
CT	computed tomography
CTLA-4	Cytotoxic T-lymphocyte-associated antigen 4
EIF1AX	Initiation Factor 1A X-linked
EMA	European Medicines Agency
FDA	Food and Drug Administration
GNA11	Guanosine Nucleotide-Binding Protein Alpha-11
GNAQ	Guanosine Nucleotide-Binding Protein Alpha-Q Gene
gp100	Glycoprotein 100
HDAC	Histone deacetylase
HLA	Human Leukocyte Antigen
HR	Hazard Ratio
ICI	Immune Checkpoint Inhibitor
LDH	lactate dehydrogenase
LITT	Laser-induced interstitial thermotherapy
MAPK	Mitogen-activated protein kinase
MEK	Mitogen-Activated Extracellular Signal-Regulated Kinase
mOS	Median overall survival
mPFS	Median progression-free survival
MRI	Magnetic Resonance Imaging
MWA	Microwave Ablation
mUM	metastatic Uveal Melanoma
NCCN	National Comprehensive Cancer Network
ORR	objective response rate
OS	overall survival
PD-1	Programmed Cell Death Protein 1
PD-L1	Programmed Death-Ligand 1
PET-CT	Positron Emission Tomography/Computed Tomography
PHP	percutaneous hepatic perfusion
PKC	Protein Kinase C
QoL	quality of life
RFA	Radiofrequency ablation
SF3B1	Splicing Factor 3B Subunit 1
SIRT	Selective Internal Radiotherapy
TACE	Transarterial Chemoembolization
TARE	transarterial radioembolization
TCR	T-cell Receptor
TMB	Tumor Mutational Burden
UM	Uveal Melanoma
VEGF	Vascular Endothelial Growth Factor
